# Supplementary material for: A scoping review protocol of existing body image guidelines for parents of youth
Source: PLoS One. 2026 Feb 13;21(2):e0343304. doi: 10.1371/journal.pone.0343304 (PMC12904369; doi:10.1371/journal.pone.0343304)
Supplement: S2 Table — (DOCX) [file pone.0343304.s002.docx]

**Supplemental Information 2: Table**

Preliminary MEDLINE Database Search Strategy

| **Search line number** | **PCC component** | **Search term(s)** | **Results** |
| --- | --- | --- | --- |
| 1 | Population (youth) | exp parent/ | 153939 |
| 2 | Population (youth) | (parent* or guardian* or caregiver* or carer* or mother* or father* or mom* or dad* or maternal or paternal or custodian*).tw,kf. | 1294657 |
| 3 | Concept (body image) | Body Image/ | 20784 |
| 4 | Concept (body image) | Body image.tw,kf. | 15984 |
| 5 | Concept (body image) | ((awareness or affect or appreciat* or assessment or attitude* or behav* or confiden* or critici* or cognition* or comment* or concern* or discrepancy or dissatisfaction or distortion or disturb* or drive for muscularity or embarrass* or esteem or envy or envious or experience? or function* or guilt or investment or perception? or pride or proud or project* or representation* or schema* or shame* or satisfaction or talk) adj2 (appearance or body)).tw,kf. | 41982 |
| 6 | Concept (body image) | ((comment* or concern* or internalization* or embarrassment or emotion* or envy or envious or guilt or pride or shame or talk) adj2 weight).tw,kf | 2806 |
| 7 | Concept (body image) | (objectification or physical appearance or physical attractiveness or physique anxiety or thinness).tw,kf. | 9030 |
| 8 | Concept (body image) | ((embarrassment or envy or shame or pride or guilt) adj2 body-related or body-focused).tw,kf. | 52 |
| 9 | Population | 1 or 2 | 1315009 |
| 10 | Concept | 3 or 4 or 5 or 6 or 7 or 8 | 66775 |
| 11 | Population and concept | 9 and 10 | 5440 |
| 12 | Limit by language and date | Limit 11 to (English language and yr=”2014-Current”) | 2967 |
