# Supplementary material for: A scoping review protocol of existing body image guidelines for parents of youth
Source: PLoS One. 2026 Feb 13;21(2):e0343304. doi: 10.1371/journal.pone.0343304 (PMC12904369; doi:10.1371/journal.pone.0343304)
Supplement: Attachment — Submitted filename: Reviewer Commets - Oct 30 2025.docx [file pone.0343304.s003.docx]

**Full Title:** A scoping review protocol of existing body image guidelines for parents of youth

**Article Type:** Study Protocol

**Overall Recommendation**

The study presents a technically sound and well-justified scoping review protocol. The rationale for synthesizing existing body image resources for parents, especially to inform the development of future sport-specific guidelines, is compelling and addresses a relevant gap in the literature. The methods are described in sufficient detail to ensure replicability and rigor. I recommend the protocol for Acceptance with Minor Revisions to address a few points of clarification regarding the scope and stakeholder engagement.

**Major Criteria Assessment**

The protocol meets all the core requirements for a Study Protocol submission to PLOS ONE:

- The background clearly establishes the need for parent-focused body image support tools. The three scoping review objectives are clearly stated and appropriate for the intended purpose of summarizing available resources.
- The protocol follows an established, eight-step methodological framework adapted for scoping reviews in sport and exercise psychology. The search strategy involving consultation with a health sciences librarian and duplicate screening/extraction steps demonstrates strong rigor.
- methods, including the databases searched and the provided search strategy (S2 Table), are detailed enough for reproduction. The plan for data extraction and content analysis (including 'critical friends' for trustworthiness) is explicitly outlined.
- The authors confirm that while screening and data extraction are in progress, the final step, recruitment and consultation with knowledge-users is not complete, confirming the study is not finished. The manuscript title contains the word "Protocol."
- The authors state that no data were generated for the protocol and that all data underlying the eventual findings will be made available upon study completion.
- The PRISMA-ScR checklist is provided as S1 Table and the protocol is prospectively registered with the Open Science Framework (OSF), which is appropriate for this type of review. Ethical approval for the subsequent focus groups is also confirmed.

**Minor Clarifications Requested (Required Revisions)**

1. **Scope Clarification on Clinical Populations (Exclusion Criteria):**

- Exclusion criterion (d) states that the protocol will exclude "clinical or treatment-seeking populations (e.g., cancer, clinical diagnosis of mental illness, medical weight management)."
- **Action Required:** Please clarify if this exclusion strictly applies to *clinically diagnosed* conditions or if it also excludes resources for parents of youth with subclinical disordered eating or body dysmorphia (which are mentioned as related conditions in the Introduction). Given the close relationship between body image concerns and disordered eating, clarifying this boundary will ensure the scope is precisely defined and avoid ambiguity during the selection process.

2. **Stakeholder Consultation Rationale (Step 1):**

- The authors note that the initial formal consultation (Step 1 in the cited framework) was not carried out due to time constraints, but this step was addressed by prior semi-structured interviews that informed the study design.
- **Action Required:** Please briefly adjust the text under the "Stakeholder Consultation" heading to state explicitly that the findings from the **prior semi-structured interviews served as the initial stakeholder consultation**, justifying the omission of the separate formal consultation meeting. This strengthens the integrity of following the adapted methodology.

3. **Search Update:**

- The protocol states that the search was conducted in January 2024. For a protocol that is currently undergoing review, it is often best practice to plan a final update search closer to the time of data analysis completion.
- **Action Required:** Please add a brief sentence to the "Search Strategy" section confirming that a final, minor update search will be conducted prior to data synthesis to capture any very recently published guidelines, ensuring the review is as current as possible upon publication of the final report.

I commend the authors for their thoughtful and detailed planning of this necessary and relevant scoping review. The inclusion of the knowledge-user focus groups (Step 8) is particularly noteworthy.
